# Granulocyte-Colony Stimulating Factor Increases Cerebral Blood Flow via a NO Surge Mediated by Akt/eNOS Pathway to Reduce Ischemic Injury

**DOI:** 10.1155/2015/657932

**Published:** 2015-06-04

**Authors:** Hock-Kean Liew, Jon-Son Kuo, Jia-Yi Wang, Cheng-Yoong Pang

**Affiliations:** ^1^Department of Medical Research, Buddhist Tzu Chi General Hospital, No. 707, Section 3, Zhongyang Road, Hualien City 970, Taiwan; ^2^Institute of Pharmacology and Toxicology, Tzu Chi University, No. 701, Section 3, Zhongyang Road, Hualien City 970, Taiwan; ^3^Graduate Institute of Medical Sciences, Taipei Medical University, No. 250, Wu-Hsing Street, Taipei 110, Taiwan; ^4^Institute of Medical Sciences, Tzu Chi University, No. 701, Section 3, Zhongyang Road, Hualien City 970, Taiwan

## Abstract

Granulocyte-colony stimulating factor (G-CSF) protects brain from ischemic/reperfusion (I/R) injury, and inhibition of nitric oxide (NO) synthases partially reduces G-CSF protection. We thus further investigated the effects of G-CSF on ischemia-induced NO production and its consequence on regional cerebral blood flow (rCBF) and neurological deficit. Endothelin-1 (ET-1) microinfused above middle cerebral artery caused a rapid reduction of rCBF (ischemia) which lasted for 30 minutes and was followed by a gradual recovery of blood flow (reperfusion) within the striatal region. Regional NO concentration increased rapidly (NO surge) during ischemia and recovered soon to the baseline. G-CSF increased rCBF resulting in shorter ischemic duration and an earlier onset of reperfusion. The enhancement of the ischemia-induced NO by G-CSF accompanied by elevation of phospho-Akt and phospho-eNOS was noted, suggesting an activation of Akt/eNOS. I/R-induced infarct volume and neurological deficits were also reduced by G-CSF treatment. Inhibition of NO synthesis by L-*N^G^*-Nitroarginine Methyl Ester (L-NAME) significantly reduced the effects of G-CSF on rCBF, NO surge, infarct volume, and neurological deficits. We conclude that G-CSF increases rCBF through a NO surge mediated by Akt/eNOS, which partially contributes to the beneficial effect of G-CSF on brain I/R injury.

## 1. Introduction

Cerebral stroke is a medical emergency which has been one of the leading causes of death and disability worldwide. Cerebral ischemia/reperfusion (I/R) injury is most frequently observed in the vascular territory of the middle cerebral artery (MCA). Therapeutic intervention by reestablishing blood flow is an essential strategy. Therapeutic benefit of thrombolytics such as recombinant tissue-plasminogen activator (rt-PA) remains limited by hemorrhagic side effects and narrow therapeutic time window [1]. A neuroprotective drug that can reestablish cerebral blood flow (CBF) without side effects is thus needed.

Granulocyte-colony stimulating factor (G-CSF) is a cytokine that can penetrate the blood brain barrier [2, 3] to exert neuroprotective effects against cerebral I/R injury [4–7]. The neuroprotective effects of G-CSF are mediated via various mechanisms, including antiapoptosis, anti-inflammation, angiogenesis, neuronal differentiation, and stem cell mobilization [5–9]. A long-term neurological recovery promoted by G-CSF from ischemic brain injury has been attributed to the enhancement of angiogenesis through endothelial proliferation to increase vascular surface area, branch points, and length [4]. Despite the long-term vasculature promoting mechanism of G-CSF, the direct effect of G-CSF on regional cerebral blood flow (rCBF) remains unclear.

Nitric oxide (NO) is a key regulator of vascular tone [10]. It is known that NO is produced by NO synthase (NOS). Several growth factors activate protein kinase Akt/PKB which can phosphorylate endothelial NOS to promote NO synthesis [11, 12]. G-CSF also exerts cardioprotective effect on myocardium during I/R injury via Akt/eNOS pathway [13]. Therefore, we tested the hypothesis that G-CSF may exert neuroprotection through activation of Akt/eNOS pathway, leading to beneficial NO release and the consequent improvement of blood flow in the ischemic brain.

## 2. Materials and Methods

### 2.1. General Procedures

All experimental protocols were approved by the Animal Care and Use Committee of the Tzu Chi University, Taiwan, in accordance with guidelines of the National Institutes of Health Guide for the Care and Use of Laboratory Animals. Animals were housed under a 12-hour light/dark cycle with free access to food and water. Utmost efforts were made to minimize the suffering and the number of animals used.

Male Sprague-Dawley rats (250 g to 350 g) were anesthetized by intraperitoneal pentobarbital (50 mg/kg, i.p., Sigma-Aldrich, St. Louis, MO, USA) or urethane (0.75 g/kg, Sigma-Aldrich). Under anesthesia, a femoral cannula (PE-50 polyethylene tube) was inserted for the monitoring of arterial pressure. The arterial pressure was recorded with MP35 amplifier (BIOPAC system, CA, USA). Body temperature was maintained automatically with a heating pad (CMA-150, CMA Microdialysis, Sweden) at 37.5 ± 0.5°C. Animal was placed in a Stoelting stereotaxic apparatus (Stoelting Co., USA) during the induction of ischemia and real-time measurement of NO level or rCBF, respectively. A midline incision of the scalp was made to expose the skull, and two burr holes were drilled for insertions of microinjection tube for ischemic induction and insertions of laser Doppler Flowmeter probe or NO-selective electrode, respectively. For evaluation of the neurological deficit, animals went through the same procedures but without the femoral cannulation.

### 2.2. Cerebral Ischemia/Reperfusion (I/R) Injury

Endothelin-1 (ET-1; Sigma-Aldrich, St. Louis, MO, USA) was used to induce transient vasoconstriction of the MCA to achieve I/R as described previously [14–16] with some modification. Briefly, 400 pmol of ET-1 in 10 *μ*L of saline was microinfused (1 *μ*L/min for 10 minutes) via a 30-gauge needle positioned in proximity to the root of MCA (stereotaxic coordinates: anterioposterior (AP) 0.0 mm, mediolateral (ML) 5.2 mm, and dorsoventral (DV) 8.0 mm relative to bregma). A >75% reduction of rCBF resulting from the ET-1 infusion served as the criterion of successful induction of ischemia.

### 2.3. Groupings

Rats were randomly assigned as groups 1 to 5: Group 1: sham control (*n* = 7), in which 10 *μ*L of saline was microinfused (1 *μ*L/min) proximate to the root of MCA at 0 min; Group 2: G-CSF (*n* = 11), in which 200 *μ*g/kg of G-CSF (Kirin Pharma. Co., Ltd, Japan), dissolved in 0.5 mL normal saline, was subcutaneously injected at 0 min without I/R induction; Group 3: I/R (*n* = 22), in which ET-1, 400 pmol in 10 *μ*L saline, was microinjected (1 *μ*L/min) proximate to the root of MCA; Group 4: I/R + G-CSF, in which G-CSF 50 *μ*g/kg (*n* = 4 for measurement of blood flow) or 200 *μ*g/kg (*n* = 28 for measurement of blood flow, NO level, neurological functions, total infarct volume, and western blot analyses), dissolved in 0.5 mL, was subcutaneously injected immediately followed by the ET-1 infusion; Group 5: NOS inhibitor + I/R + G-CSF (*n* = 17), in which NOS inhibitor L*-N* ^*G*^-Nitroarginine Methyl Ester (L-NAME; Sigma-Aldrich, St. Louis, MO, USA) 30 mg/kg, dissolved in 0.5 mL saline, was intraperitoneally administered 30 minutes prior to I/R + G-CSF administration. Rats had free access to food and water before the experiments.

### 2.4. Real-Time Measurement of Regional Cerebral Blood Flow (rCBF)

The rCBF was monitored with MNP 110XP probe (Oxford Optronix, UK) inserted during surgery and connected to a Laser Doppler Blood Flow Perfusion Monitor 403A (OxyFlo 2000, Oxford Optronix, UK). The probe was placed in the striatum at AP 0.0 mm, LM 3.0 mm, and DV 5.0 mm. The rCBF data of the baseline control period were normalized by averaging rCBF values over a 10-minute time period before drug administration by using the following formula: percentage change = [(df)/dF]*∗*100, where df is the flow after injection and dF is the mean flow during the 10-minute baseline control period.

### 2.5. Real-Time Measurement of Regional NO Concentration

Regional NO levels were monitored with a NO-selective sensor probe that consisted of porphyrin-electroplated, Nafion-coated, and carbon fiber electrodes (INC-020, Inter Medical, Japan) connected to a NO detector (IMN-101, Inter Medical, Japan) as described previously [17]. The electrodes, immersed in a small chamber filled with 0.1 M phosphate-buffered saline (PBS, pH 7.4), had been calibrated for sensitivity and selectivity by being exposed to a graded series of various concentrations (10^−3^, 10^−4^, 10^−5^, and 10^−6^ M) of the stable NO donor standard,* S*-nitroso-*N*-acetyl-DL-penicillamine (SNAP; Doujin, Kumamoto, Japan) [18]. The calibrated NO-selective electrodes were stereotaxically placed in the striatum at AP 0.0 mm, ML 3.0 mm, and DV 5.0 mm. The relative regional NO values of the baseline control period were normalized by averaging striatal NO voltage values over a 10-minute period before drug administrations.

### 2.6. Western Blot Analysis

Rats were decapitated under deep anesthesia at 0, 15, and 30 minutes and 1 hour after various treatments. The brains were rapidly removed and the ipsilateral striatal was separated and rapidly frozen in liquid nitrogen and stored at −80°C until further analysis. Protein samples (*n* = 4 for each group) for Western blot were performed as described previously with some modifications [2]. Briefly, equal amounts of protein (80 *μ*g) were loaded onto 8%-sodium dodecyl sulfate-polyacrylamide gel electrophoresis (SDS-PAGE) gel and electrophoresed, followed by blotting of the protein onto a polyvinylidene difluoride (PVDF) membrane (Millipore, Bedford, MA, USA), which were then blocked with 5% nonfat milk in 0.05% Tween-Tris-buffered saline. The membrane was then probed with primary antibodies overnight at 4°C with gentle rotation. The primary antibodies and concentrations used were as follows: phospho-eNOS (Ser 1177) (Cell Signaling, USA, 1 : 500), eNOS (Upstate, USA, 1 : 1000), phospho-nNOS (Ser 1416) (Upstate, USA, 1 : 500), nNOS (Upstate, USA, 1 : 2000), phospho-Akt (Ser 472/473) (BD, USA, 1 : 1000), Akt (BD, USA, 1 : 2000), and *β*-actin (BD, USA, 1 : 10000). Following washing and incubation with the respective secondary antibodies (Chemicon, USA, 1 : 2000;) for 1 hour at room temperature, the membranes then reacted with chemiluminescent ECL Plus Western blotting detection system (Amersham Biosciences, UK). The bands were visualized by exposure to X-ray films (Kodak, USA) and developed later. Intensities of bands were quantified with a densitometric analysis system (GS-800 Calibrated Densitometer, Bio-Rad, Hercules, CA) and calculated as the optical density × area of band.

### 2.7. Measurement of Infarct Volume

Rats were decapitated under deep anesthesia at 24 hours after I/R. Brains were removed and the forebrain was sliced into 1 mm thick coronal sections and stained with 2% 2,3,5-triphenyltetrazolium (TTC; Sigma, MO, USA) for 20 minutes at 37°C in the dark. The sections were photographed and the unstained area (i.e., the infarct area) was quantitated using Image J (NIH, USA). These sections were also used for verification of correct positioning of the ET-1 infusion tube, laser Doppler probe, or NO selective sensor probe.

### 2.8. Neurological Deficit Testing

Modified Neurological Severity Score (mNSS) was evaluated before (0) and on 1, 2, 3, 7, and 14 days after I/R in I/R, I/R + G-CSF and L-NAME + I/R + G-CSF, respectively. Neurological function is graded on a scale of 0–18 (normal score, 0; maximal deficit score, 18). The mNSS is a composite of motor, sensory, reflex, and balance tests [19].

### 2.9. Statistical Analysis

All values (regional blood flow, NO expression, and total infarct volume) are presented as means S.E.M. and analyzed by Prism software for Student's *t*-test. The statistical comparisons among multiple groups were made using one way ANOVA followed by Bonferroni correction. In all instances, *n* refers to the number of animals in a particular group. A *P* value of less than 0.05 was considered statistically significant.

## 3. Results and Discussion

### 3.1. G-CSF Accelerated Recovery of rCBF following Ischemia

**Figure 1:**
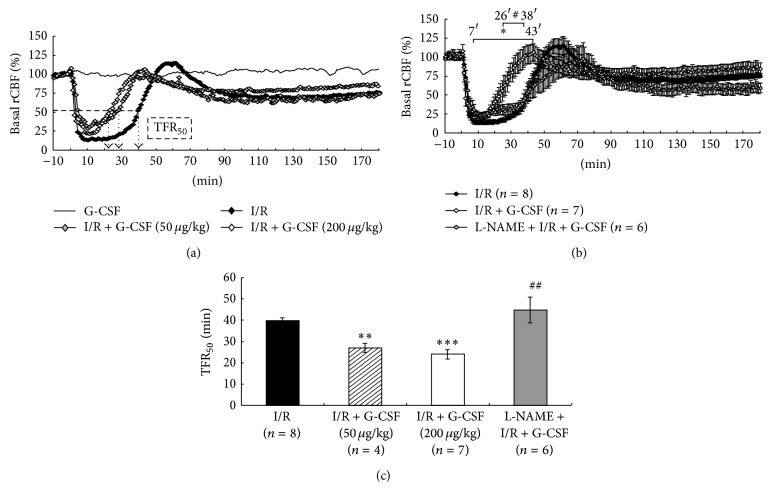
Time course of real-time rCBF in rat striata. Representative tracing of rCBF in (a) shows that administration of 50 or 200 *μ*g/kg G-CSF (I/R + G-CSF) significantly accelerated the recovery of striatal rCBF. Further real-time analysis of rCBF in rats with I/R, I/R + G-CSF, and L-NAME + I/R + G-CSF within 3 hours (b) shows that 200 *μ*g/kg G-CSF shortened the recovery time from 43 minutes to as early as 7 minutes (^*∗*^ *P* < 0.05) as compared with I/R. L-NAME pretreatment (L-NAME + I/R + G-CSF) significantly blunted the effect of G-CSF in early recovery of rCBF (from 26 minutes to 38 minutes, ^#^ *P* < 0.05) as compared with I/R + G-CSF. (c) Statistical results of TFR_50_ showing that administration of G-CSF (both 50 *μ*g/kg and 200 *μ*g/kg) significantly accelerated TFR_50_. 200 *μ*g/kg G-CSF treatment promoted faster TFR_50_ (24.0 ± 2.2 minutes, ^*∗∗∗*^ *P* < 0.001) than 50 *μ*g/kg G-CSF treated rats. 50 *μ*g/kg G-CSF treated rats showed faster TFR_50_ (27.0 ± 2.0 minutes, ^*∗∗*^ *P* < 0.01) as compared with the I/R rats (39.9 ± 1.2 minutes). L-NAME-pretreatment (L-NAME + I/R + G-CSF) blunted the shortening of TFR_50_ (44.8 ± 6.1 minutes, ^##^ *P* < 0.01) as compared with I/R + G-CSF (200 *μ*g/kg) animals. Data are presented as mean percentage changes of basal rCBF ± S.E.M.

Intracerebral infusion of ET-1 to the proximity of MCA reduced rCBF immediately by 75% of the basal within 5 minutes (resulting ischemia) and maximally by 86.7% within 8.1 ± 0.6 minutes (I/R, *n* = 8, Figure 1(b)). The blood flow recovered gradually (reperfusion): the time for flow recovery to 50% of the baseline (TFR_50_) was 39.9 ± 1.2 minutes (I/R, *n* = 8, Figure 1(c)). G-CSF (50 *μ*g/kg or 200 *μ*g/kg) subcutaneously given at the onset of ischemia shortened the TFR_50 _resulting in an earlier reperfusion (Figures 1(a) and 1(c)). Since 200 *μ*g/kg G-CSF was more effective in reducing the TFR_50_ as compared to the lower dose (50 *μ*g/kg), it was adopted for subsequent experiments (Figures 1(a) and 1(c)). G-CSF treatment significantly accelerated the recovery of the rCBF during the ischemic period (7 minutes to 43 minutes after the onset of ischemia, Figure 1(b), *P* < 0.05) and induced an early reperfusion as evidenced by the shorter TFR_50_ (Figure 1(c); I/R + G-CSF (24.0 ± 2.2 minutes) versus I/R (39.9 ± 1.2 minutes), *P* < 0.001). The basal systemic blood pressure was 101.6 ± 3.0 mmHg (*n* = 34), and no significant differences existed between the I/R and I/R + G-CSF rats (data not shown). These results indicated an accelerated recovery of blood flow by G-CSF.

### 3.2. L-NAME Abolished the Flow Recovery by G-CSF during I/R

Ample evidence suggests that NO production from eNOS is neuroprotective [20–22], especially in maintaining rCBF [20]. Pretreatment with L-NAME, a NOS inhibitor, significantly abolished the effect of G-CSF in shortening of the rCBF during the ischemic period (at 26 to 38 minutes after the onset of ischemia, Figure 1(b), *P* < 0.05) and reversed the TFR_50_ from 24.0 ± 2.2 minutes (I/R + G-CSF 200 *μ*g/kg) to 44.8 ± 6.1 minutes (L-NAME + I/R + G-CSF) (Figure 1(c), *P* < 0.01). Control animals with saline, G-CSF, or L-NAME alone had no significant effect on rCBF (data not shown). These results indicated that NO produced by NOS might participate in the effect of G-CSF on rCBF during I/R.

### 3.3. The G-CSF Enhancement of Ischemia-Induced NO Surge Was Attenuated by L-NAME

**Figure 2:**
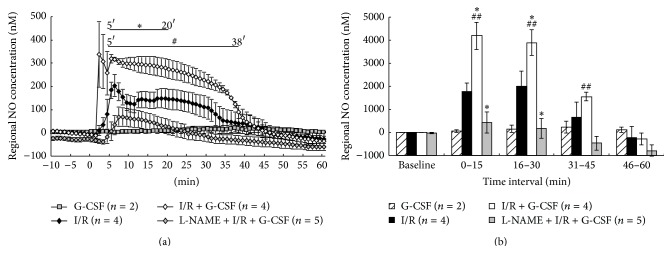
Real-time regional nitric oxide (NO) in striatal tissue of rats. (a) I/R induced rapid and sharp NO release (I/R). G-CSF treatment (I/R + G-CSF (200 *μ*g/kg)) enhanced a marked NO increase (from 5 to 20 minutes, ^*∗*^ *P* < 0.05) as compared to the I/R group. L-NAME pretreatment (L-NAME + I/R + G-CSF) significantly reduced the striatal NO release (from 5 to 38 minutes, ^#^ *P* < 0.05) as compared to the I/R + G-CSF group. (b) Cumulative real-time regional NO concentration (nM) per 15-minute interval shows that the regional NO of I/R + G-CSF increased markedly at both the 0–15- and 16–30-minute intervals (^*∗*^ *P* < 0.05) as compared to the I/R group. The L-NAME pretreatment (L-NAME + I/R + G-CSF) significantly reduced the regional NO concentration at both the 0–15- and 16–30-minute intervals as compared with I/R (^*∗*^ *P* < 0.05) or I/R + G-CSF (^##^ *P* < 0.01). Data are presented as mean of regional NO concentration ± S.E.M. (nM).

We further measured the changes of NO by real-time NO detection in the striatal tissue via a NO-selective sensor probe coupled with an* in vivo* voltammetry [17]. Regional NO increased sharply (NO surge) as soon as ischemia occurred, lasted for 35 minutes, and decreased rapidly to the baseline (Figure 2(a)). I/R + G-CSF treatment enhanced the ischemia-induced NO surge within the 5- to 20-minute period (Figure 2(a), *P* < 0.05). G-CSF also significantly increased cumulative regional NO concentration during the 0–15- and 16–30-minute periods (*P* < 0.05), but not 31–45- and 46–60-minute periods (Figure 2(b)). As compared with I/R + G-CSF group, pretreatment with L-NAME abolished NO surge induced by G-CSF within 5 to 38 minutes (Figure 2(a), *P* < 0.05).

These findings might indicate a new mechanism by which G-CSF reduces ischemic damage by promoting early recovery of rCBF through a fast production of NO, the NO surge. NO in turn will dilate cerebral blood vessels and improve collateral blood flow (striatal blood flow).

### 3.4. The G-CSF Enhancement of Ischemia-Induced NO Surge Was Mediated by Activation of Akt/eNOS Pathway

**Figure 3:**
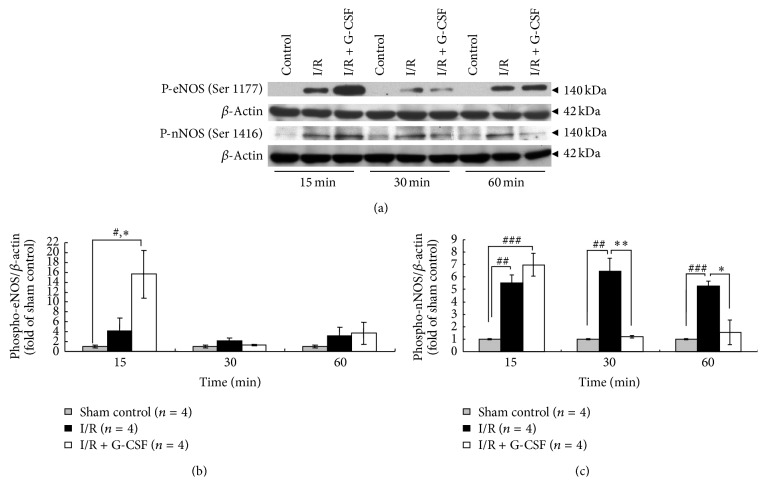
Activation of eNOS and nNOS in rats' striatal tissues during ischemia. (a) Representative changes of striatal phospho-eNOS (Ser1177) and phospho-nNOS (Ser1416) at 15, 30, and 60 minutes after ischemia in normal (sham control), I/R, and I/R + G-CSF treated rats. *β*-actin was loaded as internal quality control and used for normalization in densitometric analyses. Quantitative densitometry analysis showing relative expression of phospho-eNOS (b) and phospho-nNOS (c) as fold of sham control. ^*∗*^ *P* < 0.05 and ^*∗∗*^ *P* < 0.01 versus I/R, ^#^ *P* < 0.05 and ^##^ *P* < 0.01 versus sham control at 15, 30, and 60 min, respectively.

**Figure 4:**
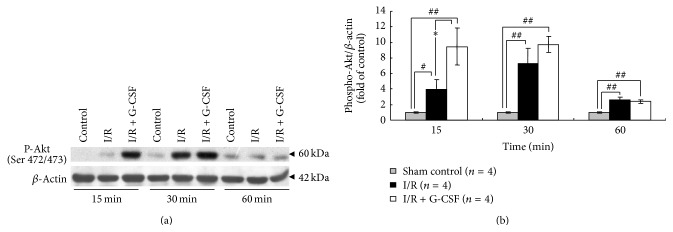
Activation of Akt in rats' striatal tissues during ischemia. (a) Representative changes of striatal phospho-Akt (Ser 472/473) at 15, 30, and 60 minutes after ischemia in normal (sham control), I/R, and I/R + G-CSF treated rats. *β*-actin was loaded as internal quality control and used for normalization in densitometric analyses. (b) Quantitative densitometry analysis showing relative expression of phospho-Akt as fold of sham control. ^*∗*^ *P* < 0.05 versus I/R, ^#^ *P* < 0.05 and ^##^ *P* < 0.01 versus sham control at each corresponding time point.

To elucidate the mechanism of G-CSF-induced NO surge in ischemic brain tissue, we delineated the Akt/eNOS signalling pathway by western blotting (Figures 3 and 4). The I/R significantly increased the levels of phosphorylated eNOS to 4.15 ± 2.54-fold at 15 minutes (Figure 3(b), *P* < 0.05). As compared with I/R group, G-CSF treatment (I/R + G-CSF) significantly increased the levels of phosphorylated eNOS to 15.66 ± 4.82-fold at 15 minutes (Figure 3(b), *P* < 0.05), and the elevation diminished at the 30- and 60-minute period in both I/R and I/R + G-CSF groups.

The I/R increased the levels of phosphorylated nNOS to 5.52 ± 0.67-, 6.46 ± 1.03-, and 5.25 ± 0.42-fold, respectively, at 15, 30, and 60 minutes (Figure 3(b), *P* < 0.01). As compared with I/R group, G-CSF treatment (I/R + G-CSF) had no significant effects on phosphorylated nNOS expression at 15 minutes but reduced the phosphorylated nNOS expression at 30 (*P* < 0.01) and 60 minutes (*P* < 0.05) (Figure 3(c)).

The I/R increased the levels of phosphorylated Akt to 3.97 ± 1.24-, 7.23 ± 1.99-, and 2.57 ± 0.39-fold, respectively, at 15, 30, and 60 minutes (Figure 4(b), *P* < 0.01). As compared with I/R group, G-CSF treatment (I/R + G-CSF) significantly increased the expression of phosphorylated Akt to 9.46 ± 2.34-fold at 15 minutes, but there were no differences at 30 and 60 minutes, respectively (Figure 4(b), *P* < 0.01). The G-CSF treatment (I/R + G-CSF) group had no significant effects on total eNOS, nNOS, and Akt expression as compared with I/R group (data not shown).

In this study, only the contributions by the constitutive NOSs (eNOS and nNOS) were considered in the G-CSF-induced enhancement of NO production, because the G-CSF effects on NO production were immediate (Figure 3). The catalytic activity of iNOS is only detectable at 12 hours after cerebral ischemia and peak after 48 hours [23]. Several studies have also shown that NO mediates neurotoxicity in primary brain cultures [24–26]; however, NO that is generated from eNOS is considered to be neuroprotective [20–22]. This tissue NO surge is probably due to the activation of eNOS rather than nNOS at initial 15 minutes (Figure 3). It is noteworthy that the timing of the NO production is very important. Early enhancement of NO concentration within 1 hour of ischemic onset is equally effective in both transient and permanent strokes, whereas later treatment is ineffective [27]. In agreement with finding by others [28–30], our results showed that early activation of both eNOS and nNOS together contributed to a sharp NO increase in the early stage of brain ischemia.

Previous study has indicated that eNOS can be activated by the serine/threonine protein kinase Akt (protein kinase B) on serine 1177, leading to the production of NO [31], which plays an important role in protecting brain tissue by augmenting rCBF after cerebral ischemia [31]. To the best of our knowledge, there has been only one report attributing the ischemia-reperfusion protective effects of G-CSF to the activation of the Akt/eNOS pathway that was in the context of I/R in an isolated perfusion rat heart model [13]. We have expanded this to the more complex neural environment and in an* in vivo* context.

### 3.5. The Neuroprotective Effects of G-CSF on Total Infarct Volume and Neurological Deficits Were Blunted by L-NAME

**Figure 5:**
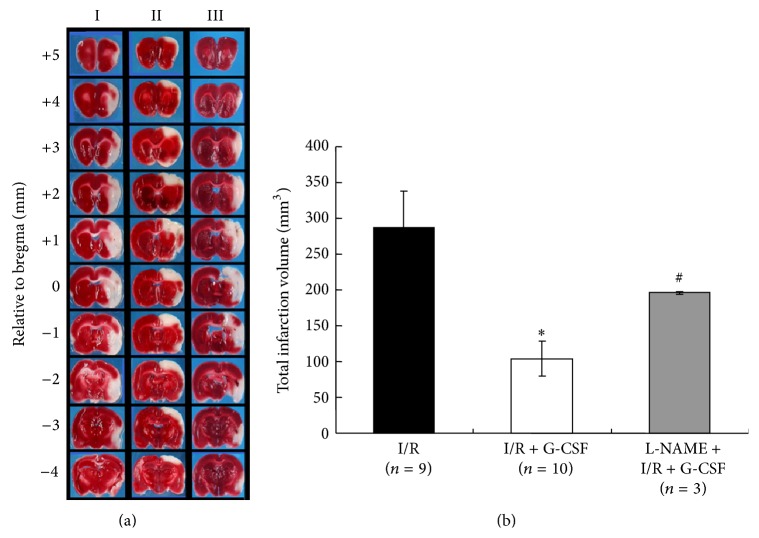
TTC staining of brain coronal sections at 24 hours after I/R. (a) TTC-stained coronal sections from I/R (Column I), I/R + G-CSF (Column II), and L-NAME + I/R + G-CSF (Column III) groups at 24 hours after I/R and (b) quantitative measurement of total infarct volumes. ^*∗*^ *P* < 0.05 versus I/R and ^#^ *P* < 0.05 versus L-NAME + I/R + G-CSF, respectively.

**Figure 6:**
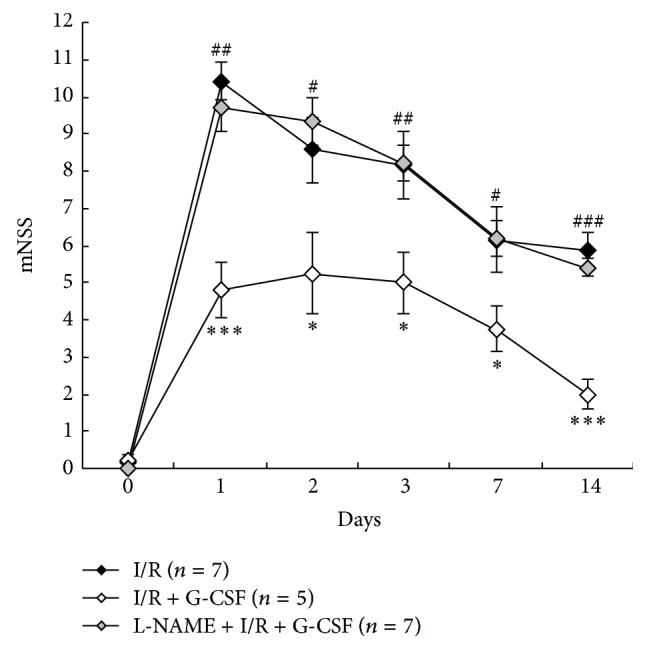
The modified Neurological Severity Score (mNSS) before I/R (0) and at 1, 2, 3, 7, and 14 days after I/R after various treatments. ^*∗*^ *P* < 0.05, ^*∗∗*^ *P* < 0.01, and ^*∗∗∗*^ *P* < 0.001 versus I/R, ^#^ *P* < 0.05, ^##^ *P* < 0.01, and ^###^ *P* < 0.001 versus I/R + G-CSF at each corresponding time points.

Figure 5(a) shows representative brain slices from I/R (I), I/R + G-CSF (II), and L-NAME + I/R + G-CSF (III) treated rats stained with 2% TTC. Measurements of total infarct volume by 2% TTC staining at 24 hours after I/R indicated a 62% reduction in total infarct volume in I/R + G-CSF group (104.15 ± 24.72 mm^3^) as compared to I/R group (287.32 ± 50.51 mm^3^) (Figure 5(b), *P* < 0.05). Neurological deficit test also indicated that G-CSF treatment (I/R + G-CSF, *n* = 4) significantly reduced the mNSS (Figure 6. *P* < 0.001 on 1 and 14 days; *P* < 0.05 on 2, 3, and 7 days after the treatment as compared to I/R group, *n* = 7). L-NAME pretreatment (L-NAME + I/R + G-CSF, *n* = 5) blunted the neuroprotective effects of G-CSF in reducing total infarct volume (Figure 5(b), *P* < 0.05 as compared to I/R + G-CSF group) and mNSS (Figure 6, *P* < 0.01 at 1 and 3 days; *P* < 0.05 at 2 and 7 days; and *P* < 0.001 at 14 days as compared to I/R + G-CSF group).

### 3.6. Does G-CSF Still Have a Chance to Serve as a Stroke Drug?

Although ample animal studies and several human trials have demonstrated the potential of G-CSF as a candidate for early stroke drug, the failure of AXIS 2 is a huge setback [32]. AXIS 2 was a European multicenter, randomized, and double-blind placebo-controlled Phase IIb trial. The aim of this trial was to show the clinical efficacy of infusing 135 *μ*g/kg body weight G-CSF over a period of 72 hr, for the treatment of acute ischemic stroke which occurred within 9 hours of symptom onset. However, G-CSF failed to meet the primary (improvement on the modified Rankin scale after 90 days) and secondary (less than a half-point difference in the NIH Stroke scale after 90 days) endpoints in AXIS 2. As discussed by the authors, the reasons for the failure of G-CSF remain obscure. As opposed to 5 consecutive subcutaneous injections of ~10 *μ*g/kg body weight/day in treating neutropenia, the extraordinary high dose of G-CSF infusion in AXIS 2 was proved to be safe in a previous IIa trial, and thus the dose might not be the cause of failure.

The decrease in mean arterial blood pressure (MAP) and increase in heart rate were unexpected in G-CSF-treated patients. The authors speculated that the decrease in MAP was caused by the direct effect of G-CSF on the vasculature with a lowering of peripheral resistance. Since NO can cause vessel dilation, it will be interesting to learn if G-CSF, when given as early as possible after stoke onset, can help in the recovery of rCBF. Our current study also shows that a NO surge that occurs immediately after stroke is beneficial. It will also be of great interest to learn if G-CSF can exert its therapeutic effect when given simultaneously in the rt-PA therapy. Thus, it is still too premature to deny the application of G-CSF in early stroke therapy.

## 4. Conclusions

G-CSF is beneficial in terms of promoting early recovery of rCBF, reduction of total infarct volume, and neurological deficit. We have provided evidence supporting enhanced NO production via the activation of Akt/eNOS pathway as the probably mediator of such beneficial effects. Taken together, our results suggest that G-CSF may have the potential as a novel therapeutic agent or part of a therapeutic regimen for acute stroke.
